# Elevated NK and T cell-associated cytokines in plasma are associated with serological response to influenza vaccination

**DOI:** 10.3389/fimmu.2025.1662942

**Published:** 2025-08-13

**Authors:** Harry Pickering, Michael A. Carlock, Monica Cappelletti, David W. Gjertson, Ted M. Ross, Elaine F. Reed

**Affiliations:** ^1^ David Geffen School of Medicine, University of California, Los Angeles, Los Angeles, CA, United States; ^2^ Center for Vaccines and Immunology, College of Veterinary Medicine, University of Georgia, Athens, GA, United States; ^3^ Florida Research and Innovation Center, Cleveland Clinic, Port Saint Lucie, FL, United States; ^4^ Department of Infectious Diseases, College of Veterinary Medicine, University of Georgia, Athens, GA, United States

**Keywords:** influenza, FluZone vaccination, chemokines, cytokines, serological immunity

## Abstract

**Introduction:**

Numerous pre-vaccination factors are known to be associated with differential responses to influenza vaccination, including age, prior infection, vaccination history, immune cell frequencies, and transcriptomic profiles. However, plasma chemokines and cytokines are relatively unexplored. Given that older individuals have generally higher levels of inflammatory molecules in circulation, termed inflammaging, and also respond poorly to vaccination, plasma immune profiles likely play a role in effective response to influenza vaccination.

**Methods:**

A cohort of 100 people were sampled pre- (Day 0) and post-vaccination (Day 7) with the inactivated, quadrivalent Fluzone construct in the autumn of 2019 (UGA4). Plasma chemokines and cytokines were quantified by 38-plex Luminex assay, with ultrasensitive quantification of additional analytes by Single Molecule Array Technology (Simoa) assay. Antibodies against individual strains of influenza and serological response to vaccination were determined by Day 0 hemagglutination inhibition (HAI) titer and change in HAI titers from Day 0 to Day 28, respectively.

**Results:**

Age was strongly associated with pre-vaccination HAI titers and differences in plasma analytes, but not changes in HAI titers post-vaccination. High plasma levels of eotaxin (CCL11) and MDC (CCL22) pre-vaccination were associated respectively with ineffective and effective serological response to vaccination. Increasing plasma levels of IFN-γ, IL-17A, and IL-15 from Day 0 to Day 7 post-vaccination were associated with effective serological response to vaccination.

**Discussion:**

In conclusion, plasma chemokines and cytokine levels prior to or in the first few days post-influenza vaccination may be predictive of serological responses to vaccination, with changes in IFN-γ, IL-17A, and IL-15 post-vaccination possibly indicative of the activation of cell-mediated immunity. These findings support the need for larger, high-resolution studies exploring the role of plasma proteomics in serological responses to influenza vaccination.

## Introduction

Despite seasonal vaccination efforts (1), influenza still causes significant global morbidity and mortality (2). This is in part driven by considerable patient-to-patient heterogeneity in vaccine efficacy (3, 4). A number of host factors that impact vaccine immunogenicity and protection have been identified (5), including age, sex, diet, and immune history. However, correlates of responsiveness to influenza vaccination are still incompletely understood.

Considerable progress has been made in understanding correlates and mechanisms of influenza vaccine efficacy in the previous two decades using a variety of systems biological tools, including multivariate measures of cellular and humoral immunity, epigenetics, metabolomics, and transcriptomics (6–8). There have also been a number of studies exploring the relationship of circulating chemokines and cytokines to influenza vaccination (9–13), a popular approach given the relative ease of measuring these analytes. Multiple inflammatory cytokines increase following vaccination, most often IFN-γ, IL-10, and TNFα. However, findings on the relationship between these circulating factors and serological response to vaccination have been inconsistent. Post-vaccination levels of IL-6 and TNFα were positively correlated with increasing hemagglutination inhibition (HAI) titers (11), while another report found no relationship for these cytokines, but instead a positive correlation of IL-10 with increasing HAI titers (12). Another study measured all three of these cytokines pre- and post-vaccination (13), finding no association with HAI titers at either timepoint. One consistent finding from these studies is that associations vary depending on the vaccine type and by influenza strain, as well as differences in study design.

As a result of these inconsistencies, further research is required to understand the value of pre- and post-vaccination chemokines and cytokines in predicting serological responses to influenza vaccination. Therefore, we sought to determine the relationship of circulating analytes with total and strain-specific serological responses to influenza vaccination. This study used a cohort of 100 participants receiving the quadrivalent Fluzone vaccination against influenza (14), part of the Center for Influenza Vaccine Research for High-Risk Populations (CIVR-HRP) program, to profile plasma analytes and their relationship to serological immunity. This work identified pre-vaccination levels and post-vaccination changes in plasma chemokines and cytokines that were correlated with serological responsiveness, including strain-specific differences.

## Materials and methods

### Cohorts of seasonal influenza vaccination

The study’s cohorts have been described in detail previously (15). Briefly, as part of an ongoing study by the University of Georgia, Athens (UGA), a total of 690 participants were recruited during five seasons between 2016 and 2020 (UGA1–5). The study procedures, informed consent, and data collection documents were reviewed and approved by the Institutional Review Board of the University of Georgia (IRB #3773). Participants received the standard dose (15 μg/antigen) split-virion (IIV) version of licensed Fluzone by Sanofi Pasteur (Swiftwater, PA, USA). Vaccine formulation was based on WHO recommendations for the Northern Hemisphere influenza seasons beginning in the fall. For the 2019–2020 season (UGA4), the strains were A/Brisbane/2018 (H1N1), A/Kansas/2017 (H3N2), B/Phuket/2013 (Yamagata lineage), and B/Colorado/2017 (Victoria lineage). Participants provided blood samples on the day of vaccination (“Day 0”) prior to vaccine administration and 7/28 days post‐vaccination (“Day 7” and “Day 28”). HAI assays were performed on the Day 0 and Day 28 blood samples against each of the vaccine strains as well as other strains. For this study, a subset of 100 participants enrolled in UGA4, who had been enrolled and vaccinated as part of UGA3, were chosen. These participants were selected so that we could explore the impact of pre-vaccination immunity, as well as provide a wide distribution of participant ages.

### Plasma analyte profiling by 38-plex Luminex

Plasma were assayed for cytokines and chemokines involved in stimulating neutrophils, antigen-presenting cells (APCs) (i.e., CXCL1, CXCL2, CXCL5, CXCL8, CCL2, CCL5, CCL20, IL-1a, IL-1b, IL-17, IFN-γ, and IFN I), T cells (GM-CSF, IFN-γ, IL-2, IL-4, IL-5, IL-13, and IL-17, among others), and B cells (IFN-γ, IL-12, IL-2, IL-4, IL-13, IL-10, and TGF-β) via 38-plex Luminex multibead arrays (Millipore Sigma, Burlington, Massachusetts) acquired on a Luminex 200 System (Luminex). Standard curves per analyte were used to define pg/mL concentrations for each sample. Plasma analytes were quantified on blood samples from Day 0 and Day 7, but not Day 28.

### Targeted, ultrasensitive plasma profiling by Simoa

To maximize the resolution for detecting changes in targeted cytokines, we utilized the ultrasensitive Single Molecule Array Technology (Simoa) immunoassay to quantify IL-1α, IL-1β, and IL-15 (Quanterix, MA, USA). The limits of detection for IL-1α, IL-1β, and IL-15 were 0.062, 0.011, and 0.003 pg/mL, respectively, by Simoa, compared with 4.8, 1.6, and 3 pg/mL, respectively, by Luminex. Standard curves per analyte were used to define pg/mL concentrations for each sample. Plasma analytes were quantified on blood samples from Day 0 and Day 7, but not Day 28.

### Statistical analysis

Influenza strain-specific HAI titers were analyzed pre-vaccination (“Day 0”) and post-vaccination (“Day 28”). Additionally, log2 fold-change in HAI titers from pre- to post-vaccination was analyzed. Analyses of post-vaccination HAI titers or changes in titers included pre-vaccination HAI titer as a covariate. Analytes with >25% of samples undetectable for an individual assay were excluded from downstream analyses. Using Luminex, 16/38 analytes were included. Using Simoa, three analytes were included. For Day 0 and Day 7 analyses, chemokine/cytokine concentrations in pg/mL were used. Additionally, log2 fold-change in concentrations from Day 0 to Day 7 was analyzed. Univariate analysis of plasma analytes with respect to HAI titers against individual strains was conducted by linear regression, adjusting for age, gender, and body mass index (BMI); see the Results section for the detailed justification of covariates. Models were considered significant if p-value ≤ 0.05.

## Results

### Age is associated with lower pre- and post-vaccination HAI titers

HAI titers against all four strains of influenza were incorporated in the UGA4 vaccine construct post-vaccination (**Table 1**). The greatest increase was observed against A/Kansas/2017, which also had the lowest pre-vaccination titer. To understand which demographic factors were associated with HAI titers and serological response to influenza vaccination, we analyzed the association between these variables and strain-specific HAI titers at Day 0 and Day 28. Age was significantly associated with pre-vaccination HAI titers against all four strains and post-vaccination titers against three out of four strains (**Table 2**, **Supplementary Figure S1**). Female participants trended toward lower pre-vaccination HAI titers against influenza B strains, significantly for B/Colorado/2017 (**Table 2**, **Supplementary Figure S2**), but there was no significant association between gender and post-vaccination titers. BMI was not associated with pre- or post-vaccination titers against three out of four strains (**Table 3**, **Supplementary Figure S3**), but there was a positive association between BMI and titers against B/Colorado/2017, significantly so post-vaccination.

**Table 1:** Participant demographics.

Demographic variable	UGA4 cohort (N = 100)
Median age (IQR)	49 (31–65)
Female gender (%)	56 (56%)
Median BMI (IQR)	27.9 (24.9–31.8)

BMI, body mass index; IQR, interquartile range.

**Table 2:** Distributions of HAI titers.

Influenza strain	Visit*	Median titer (IQR)	Age		Female gender		BMI	
			Linear coefficient (SE**)	P-value	Linear coefficient (SE**)	P-value	Linear coefficient (SE**)	P-value
**A/Brisbane/2018 (H1N1)**	Day 0	20 (10–40)	−0.035 (0.006)	1.40E−08	−0.295 (0.232)	0.207	−0.003 (0.016)	0.838
**A/Brisbane/2018 (H1N1)**	Day 28	40 (20–80)	−0.023 (0.006)	1.10E−04	−0.122 (0.221)	0.584	0.019 (0.015)	0.223
**A/Kansas/2017 (H3N2)**	Day 0	5 (5–10)	−0.01 (0.004)	0.03	0.181 (0.16)	0.26	−0.012 (0.011)	0.273
**A/Kansas/2017 (H3N2)**	Day 28	40 (20–160)	0.001 (0.007)	0.94	0.279 (0.257)	0.281	0.015 (0.018)	0.385
**B/Phuket/2013 (Yamagata)**	Day 0	40 (20–160)	−0.035 (0.006)	5.10E−07	−0.374 (0.256)	0.147	−0.019 (0.018)	0.279
**B/Phuket/2013 (Yamagata)**	Day 28	80 (40–320)	−0.039 (0.006)	1.70E−09	−0.1 (0.248)	0.687	0.011 (0.017)	0.504
**B/Colorado/2017 (Victoria)**	Day 0	20 (10–40)	−0.014 (0.007)	0.041	−0.504 (0.232)	0.032	0.025 (0.016)	0.117
**B/Colorado/2017 (Victoria)**	Day 28	80 (40–80)	−0.012 (0.006)	0.031	−0.26 (0.198)	0.193	0.033 (0.013)	0.014

HAI, hemagglutination inhibition; BMI, body mass index.

*Day 0 = pre-vaccination and Day 28 = post-vaccination.

**SE, standard error.

**Table 3:** Plasma analyte distributions.

Analyte	Median pg/mL (IQR)	Below LOD* (%)	Age		Female gender		BMI	
			Linear coefficient (SE**)	P-value	Linear coefficient (SE)	P-value	Linear coefficient (SE)	P-value
EGF	14.1 (8–21.5)	25.5	0.003 (0.004)	0.457	0.102 (0.128)	0.427	−0.005 (0.008)	0.541
Eotaxin	665.1 (456.8–895.3)	0.0	0.006 (0.003)	0.045	−0.431 (0.098)	2.00E−05	−0.031 (0.007)	7.10E−06
FGF.2	60.9 (37.5–95.7)	24.0	−0.002 (0.004)	0.679	−0.178 (0.136)	0.193	0.005 (0.01)	0.635
Flt.3L	18.5 (8.6–34.4)	65.5	0.024 (0.006)	1.00E−04	0.333 (0.203)	0.106	0.008 (0.014)	0.546
Fractalkine	41.8 (19.8–66.5)	39.5	0.005 (0.005)	0.311	−0.024 (0.161)	0.884	0.02 (0.012)	0.107
G.CSF	21.3 (8.8–70.8)	56.5	0.012 (0.009)	0.181	0.055 (0.298)	0.854	0.032 (0.022)	0.139
GM.CSF	4.7 (3–10.8)	29.0	0.004 (0.005)	0.463	0.155 (0.191)	0.419	0.001 (0.013)	0.959
GRO	287.8 (162.3–484.3)	1.0	0.005 (0.004)	0.166	0.197 (0.138)	0.155	0.002 (0.01)	0.866
IFN.a2	9.7 (5.4–26)	45.5	0.001 (0.006)	0.931	−0.116 (0.2)	0.564	0.052 (0.016)	0.001
IFN.g	7.1 (3.5–11.2)	21.0	−0.001 (0.005)	0.922	0.083 (0.188)	0.659	−0.006 (0.012)	0.613
IL.10	10.2 (5.2–23.6)	11.0	0.009 (0.005)	0.038	0.053 (0.165)	0.747	0.027 (0.011)	0.013
IL.12.p40	7.4 (3.2–19.7)	42.0	0.018 (0.006)	0.005	−0.305 (0.234)	0.194	−0.005 (0.015)	0.739
IL.12.p70	3.4 (1.3–8.5)	46.0	0.004 (0.008)	0.563	−0.329 (0.267)	0.221	0.034 (0.017)	0.051
IL.13	22.6 (5.3–70.9)	58.5	0.01 (0.009)	0.279	0.532 (0.365)	0.15	0.027 (0.025)	0.282
IL.15	8.6 (7.1–10.6)	0.5	0 (0.001)	0.727	0.01 (0.05)	0.846	0 (0.003)	0.975
IL.17A	2.4 (1.4–5.2)	23.5	−0.002 (0.005)	0.772	−0.195 (0.192)	0.313	0.002 (0.015)	0.873
IL.1a	208.1 (98.1–537.4)	68.0	0.007 (0.009)	0.42	0.132 (0.394)	0.738	0.006 (0.024)	0.802
IL.1a	0.5 (0.1–1)	11.0	−0.017 (0.007)	0.021	0.129 (0.263)	0.623	−0.031 (0.017)	0.075
IL.1b	0.8 (0–2.4)	44.5	−0.004 (0.014)	0.765	−0.129 (0.496)	0.795	−0.045 (0.029)	0.124
IL.1RA	65.2 (32.4–224.5)	4.0	0.002 (0.005)	0.708	0.359 (0.177)	0.044	0.025 (0.012)	0.041
IL.2	0.9 (0.4–2.1)	73.5	−0.005 (0.011)	0.631	−0.26 (0.367)	0.482	0.04 (0.021)	0.063
IL.3	1.4 (1.3–1.5)	98.0	−0.002 (0.003)	0.504	−0.096 (0.074)	0.325	0.001 (0.004)	0.868
IL.4	567.5 (255–1,203)	67.5	0.005 (0.009)	0.631	0.288 (0.407)	0.481	−0.012 (0.024)	0.609
IL.5	5.7 (1.9–15.1)	60.5	0.003 (0.01)	0.794	1.029 (0.362)	0.006	0.002 (0.027)	0.942
IL.6	121.3 (41.4–300.3)	63.5	0.006 (0.009)	0.502	0.841 (0.351)	0.019	−0.002 (0.024)	0.938
IL.7	12.3 (5.6–50.5)	80.5	0.014 (0.01)	0.171	−0.026 (0.396)	0.947	0.029 (0.019)	0.14
IL.8	18.4 (6.5–41.4)	2.5	−0.001 (0.005)	0.819	0.409 (0.18)	0.024	−0.001 (0.013)	0.962
IL.9	9.9 (3.8–31.8)	60.0	0.009 (0.009)	0.352	0.288 (0.383)	0.455	0.031 (0.025)	0.212
IP.10	371.9 (277.8–591.8)	0.0	0.014 (0.002)	5.20E−09	0.151 (0.085)	0.076	0.011 (0.006)	0.072
MCP.1	573.9 (459–725.2)	0.0	0.006 (0.002)	2.90E−04	−0.203 (0.054)	2.20E−04	0.004 (0.004)	0.249
MCP.3	115.3 (59.1–182.9)	59	0.006 (0.005)	0.239	0.382 (0.204)	0.065	0.006 (0.014)	0.658
MDC	682.5 (559.1–844)	0	0.001 (0.001)	0.628	0.174 (0.051)	7.80E−04	0.005 (0.004)	0.17
MIP.1a	4 (3.3–5.7)	66.5	0.008 (0.007)	0.309	−0.191 (0.259)	0.462	0.043 (0.014)	0.004
MIP.1b	23.3 (15.3–32.2)	0.0	0.008 (0.002)	2.00E−04	−0.126 (0.073)	0.086	0.022 (0.005)	6.70E−06
sCD40L	323.4 (159.9–594.5)	0.0	−0.003 (0.004)	0.467	0.069 (0.147)	0.638	−0.001 (0.01)	0.917
TGF.a	5 (3.6–8.1)	60.5	0.013 (0.006)	0.03	−0.093 (0.215)	0.665	0.006 (0.012)	0.62
TNF.a	17.7 (12.2–26.8)	0.0	0.011 (0.002)	1.00E−07	−0.011 (0.076)	0.883	0.017 (0.005)	0.001
TNF.b	164.2 (65.2–403.5)	63.5	0.001 (0.01)	0.953	0.559 (0.398)	0.164	0 (0.027)	0.996
VEGF	55.1 (28.7–93.4)	48.5	0.014 (0.006)	0.021	−0.05 (0.21)	0.812	0.014 (0.015)	0.366

BMI, body mass index.

*LOD, limit of detection; **SE, standard error.

### Plasma analytes are most strongly associated with age

Of the 39 plasma chemokines and cytokines measured in this study, 16 (41%; eotaxin, FGF-2, GRO, IFN-γ, IL-10, IL-15, IL-17A, IL-1α, IL-1Rα, IL-8, IP-10, MCP-1, MDC, MIP-1β, sCD40L, and TNFα) were above the limit of detection in >75% of participants across all study timepoints (**Table 3**). Of these analytes, seven were significantly associated with age. The association was positive for six out of seven, and only IL-1a levels were significantly negatively associated with age. Additionally, five analytes were significantly associated with gender and BMI. When median levels of all analytes were clustered together (**Supplementary Figure S4**), there was some evidence of older and female participants clustering together with higher levels of multiple analytes. Due to the observed association of both HAI titers and plasma analyte levels with age, gender, and BMI, and previously reported impact of these demographic factors on response to influenza vaccination, all three were included as covariates in our analyses.

### Age correlates with HAI titers but not changes in titers post-vaccination

**Figure 1:**
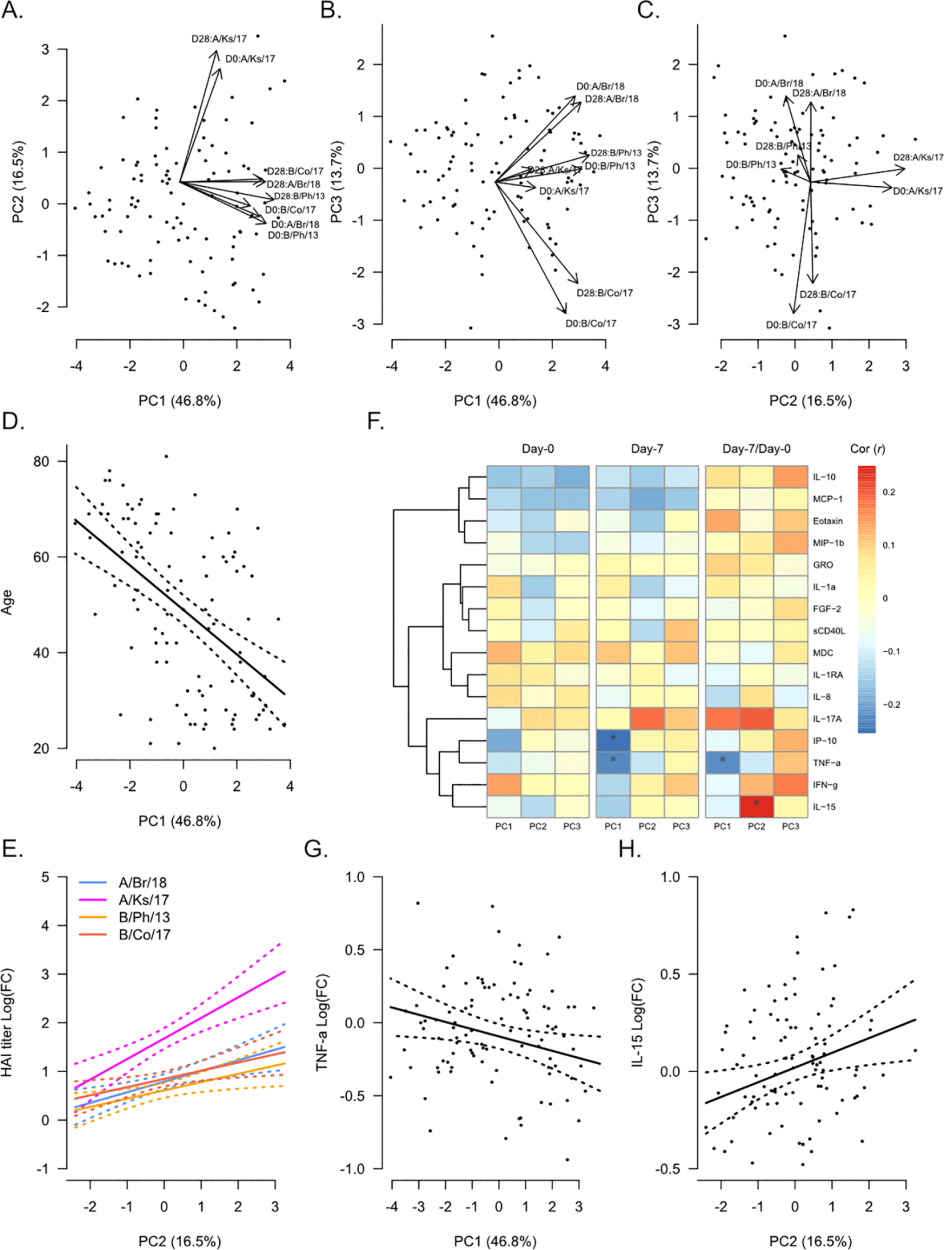
Global analysis of hemagglutination inhibition (HAI) titers in correlation with participant demographics and plasma analytes. Principal component analysis (PCA) was performed on Day 0 (pre-vaccination) and Day 28 post-vaccination HAI titers against A/Brisbane/2018 (A/Br/18, blue), A/Kansas/2017 (A/Ks/17, pink), B/Phuket/2013 (B/Ph/13, orange), and B/Colorado/2017 (B/Co/17, red). Participant eigenvalues and variable loadings are shown for principal component 1 (PC1) vs. PC2 **(A)**, PC1 vs. PC3 **(B)**, and PC2 vs. PC3 **(C)**. **(D)** Visualization of the relationship between participant age and PC1. The solid line shows the linear regression of age on PC1, and the dotted lines show 95% confidence intervals. **(E)** Visualization of the relationship between fold-change in HAI titers from Day 0 to Day 28 and PC2. Solid lines show the linear regression of age on PC1, and dotted lines show 95% confidence intervals. **(F)** Pearson’s correlation (*r*) between each PC and plasma analytes measured at Day 0 and Day 7 and their fold-change from Day 0 to Day 7 (“Day 7/Day 0”). Significant correlations are indicated as follows: *p ≤ 0.05. Visualization of the relationship of fold-change in TNFα **(G)** and IL-15 **(H)** with PC1 and PC2, respectively. Solid lines show the linear regression of age on PC1, and dotted lines show 95% confidence intervals.

To evaluate the global relationship of participant demographics and plasma analyte levels with influenza HAI titers, we performed principal component analysis (PCA) on strain-specific HAI titers from Day 0 and Day 28 (**Figures 1A–C**). The first principal component (PC), which accounted for approximately 47% of the total variance, reflected increasing HAI titers against all strains (**Figure 1A**). Age was significantly negatively correlated with PC1 (Pearson’s *r* = −0.51, p = 0.6 × 10^−7^, **Figure 1D**), reflecting decreasing HAI titers with age. Additionally, age was weakly negatively correlated with PC3 (*r* = −0.22, p = 0.03). The second PC, which accounted for ~17% of the total variance, showed some association with serological response to vaccination, with higher values on this PC for Day 28 titers compared with Day 0 (**Figures 1A, C**). In support of this, fold-change in HAI titer from Day 0 to Day 28 was significantly positively correlated with PC2 for all strains (**Figure 1E**). Gender was also significantly, but weakly, correlated with PC2 (*r* = 0.20, p = 0.04). The third PC, which accounted for ~14% of the total variance, appeared to differentiate participants based on strain-specific HAI titers (**Figures 1B, C**). The highest values were associated with increased HAI titers against A/Brisbane/2018, while lower values were associated with increased HAI titers against B/Colorado/2017.

We next determined the correlation between plasma analyte levels and each PC (**Figure 1F**) using analyte levels at Day 0 and Day 7 and the fold-change from Day 0 to Day 7 (“Day 7/Day 0”). No plasma analytes measured at Day 0 were significantly correlated with any PC. However, the inflammatory proteins IP-10 (CXCL10, *r* = −0.25, p = 0.01) and TNFα (*r* = −0.23, p = 0.02) measured at Day 7 were negatively correlated with PC1. Fold-change in TNFα from Day 0 to Day 7 was also negatively correlated with PC1 (**Figure 1G**, *r* = −0.23, p = 0.03). We also observed a significant positive correlation between fold-change in IL-15 from Day 0 to Day 7 and PC2 (**Figure 1H**, *r* = 0.25, p = 0.01).

### Plasma analytes pre- and early post-vaccination are associated with serological response to vaccination

**Figure 2:**
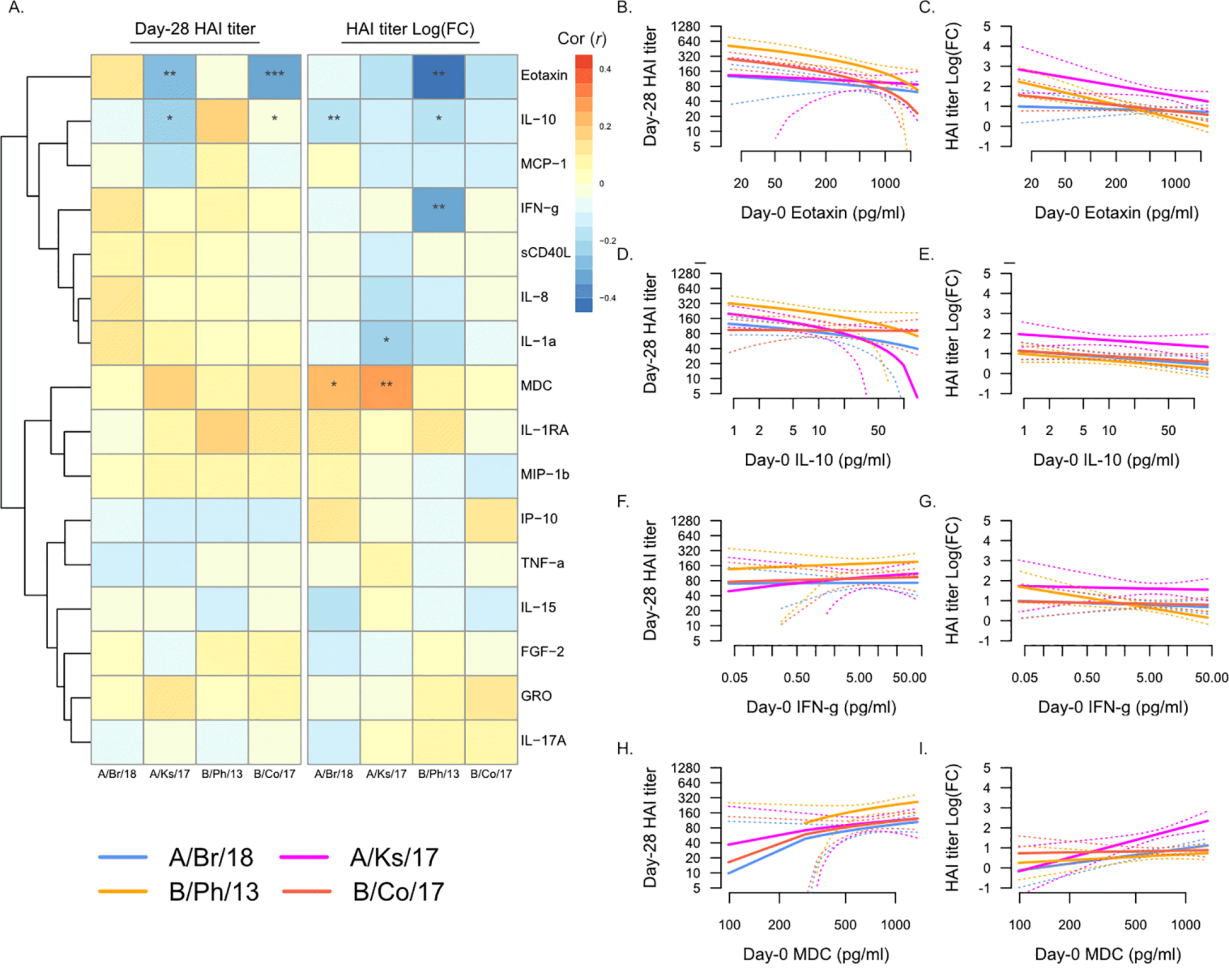
Day 0 pre-vaccination plasma analytes and post-vaccination hemagglutination inhibition (HAI) titers. **(A)** Pearson’s correlation (*r*) of Day 28 post-vaccination HAI titers (“Day 28 HAI titer”) and fold-change in HAI titers post-vaccination [“HAI titer Log(FC)”] with plasma analytes measured at Day 0. Significant correlations are indicated as follows: *p ≤ 0.05, **p ≤ 0.01, and ***p ≤ 0.001. Strains are shown as follows: A/Brisbane/2018 (A/Br/18, blue), A/Kansas/2017 (A/Ks/17, pink), B/Phuket/2013 (B/Ph/13, orange), and B/Colorado/2017 (B/Co/17, red). Visualization of the relationship of Day 28 HAI titers and fold-change in HAI titers from Day 0 to Day 28 and eotaxin **(B, C)**, IL-10 **(D, E)**, IFN-γ **(F, G)**, and MDC **(H, I)**. Solid lines show the linear regression of age on PC1, and dotted lines show 95% confidence intervals.

We next determined the association between Day 0 pre-vaccination plasma analyte levels and Day 28 post-vaccination HAI titers against individual strains of influenza (**Figure 2**). Overall, plasma analyte levels were more strongly associated with fold-change in HAI titer from Day 0 to Day 28, compared to Day 28 titers (**Figure 2A**). Eotaxin (CCL11) levels were negatively correlated with Day 28 titers against A/Kansas/2017 and B/Colorado/2017 (**Figure 2B**), as well as fold-change in titers for all four vaccine strains (**Figure 2C**), significantly so for B/Phuket/2013. Similarly, IL-10 levels were negatively correlated with Day 28 titers against A/Kansas/2017 and B/Colorado/2017 (**Figure 2D**), as well as fold-change in titers for all four vaccine strains (**Figure 2E**), significantly so for A/Brisbane/2018 and B/Phuket/2013. IFN-γ levels were also significantly negatively correlated with fold-change in titers against B/Phuket/2013 (**Figures 2F, G**). In contrast, MDC (CCL22) levels were positively correlated with fold-change in titers for all four vaccine strains (**Figures 2H, I**), significantly so for A/Brisbane/2018 and A/Kansas/2017.

**Figure 3:**
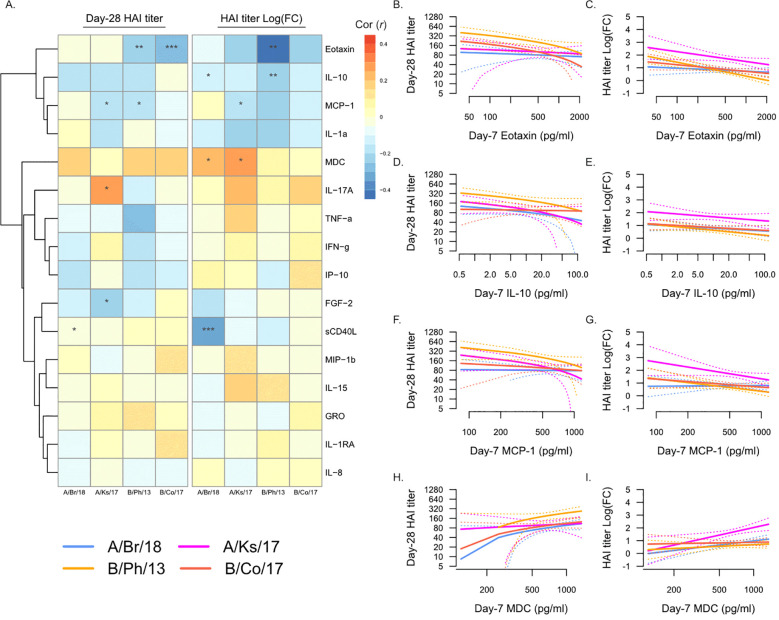
Day 7 post-vaccination plasma analytes and post-vaccination hemagglutination inhibition (HAI) titers. **(A)** Pearson’s correlation (*r*) of Day 28 post-vaccination HAI titers (“Day 28 HAI titer”) and fold-change in HAI titers post-vaccination [“HAI titer Log(FC)”] with plasma analytes measured at Day 7. Significant correlations are indicated as follows: *p ≤ 0.05, **p ≤ 0.01, and ***p ≤ 0.001. Strains are shown as follows: A/Brisbane/2018 (A/Br/18, blue), A/Kansas/2017 (A/Ks/17, pink), B/Phuket/2013 (B/Ph/13, orange), and B/Colorado/2017 (B/Co/17, red). Visualization of the relationship of Day 28 HAI titers and fold-change in HAI titers from Day 0 to Day 28 and eotaxin **(B, C)**, IL-10 **(D, E)**, MCP-1 **(F, G)**, and MDC **(H, I)**. Solid lines show the linear regression of age on PC1, and dotted lines show 95% confidence intervals.

We next asked whether the levels of plasma analytes at Day 7 post-vaccination were associated with post-vaccination HAI titers (**Figure 3**). Strikingly, many of the associations between Day 0 plasma analytes and post-vaccination titers were maintained at Day 7 (**Figure 3A**), most notably the negative association with eotaxin (**Figures 3B, C**) and IL-10 (**Figures 3D, E**) and the positive association with MDC (**Figures 3H, I**). In addition, Day 7 MCP-1 (CCL2) levels were negatively associated with serological response to vaccination (**Figures 3F, G**), both Day 28 titers and fold-change in titers against A/Kansas/2017 and Day 28 titers only against B/Phuket/2013. The levels of sCD40L were negatively associated with fold-change in titers against A/Brisbane/2018, while IL-17A was weakly positively associated with Day 28 titers against A/Kansas/2017.

### Increasing NK and T cell-associated cytokine levels post-vaccination are associated with serological response to vaccination

**Figure 4:**
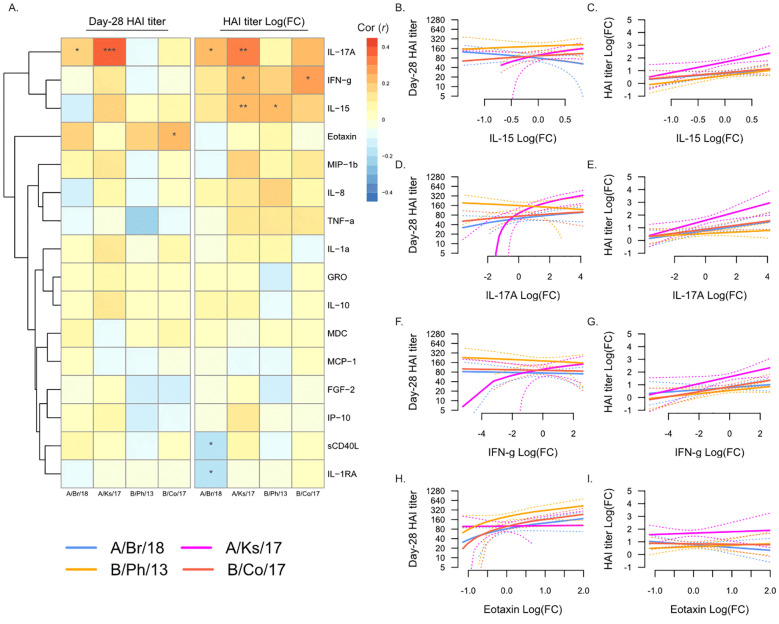
Changes in plasma analytes post-vaccination and post-vaccination hemagglutination inhibition (HAI) titers. **(A)** Pearson’s correlation (*r*) of Day 28 post-vaccination HAI titers (“Day 28 HAI titer”) and fold-change in HAI titers post-vaccination [“HAI titer Log(FC)”] with fold-change in plasma analytes from Day 0 to Day 7. Significant correlations are indicated as follows: *p ≤ 0.05, **p ≤ 0.01, and ***p ≤ 0.001. Strains are shown as follows: A/Brisbane/2018 (A/Br/18, blue), A/Kansas/2017 (A/Ks/17, pink), B/Phuket/2013 (B/Ph/13, orange), and B/Colorado/2017 (B/Co/17, red). Visualization of the relationship of Day 28 HAI titers and fold-change in HAI titers from Day 0 to Day 28 and IL-15 **(B, C)**, IL-17A **(D, E)**, IFN-γ **(F, G)**, and eotaxin **(H, I)**. Solid lines show the linear regression of age on PC1, and dotted lines show 95% confidence intervals.

We next determined whether changes in any plasma analytes from Day 0 to Day 7 post-vaccination were associated with serological response to vaccination (**Figure 4**). As described above, changes in plasma analyte levels were more strongly associated with fold-change in HAI titer from Day 0 to Day 28, compared to Day 28 titers (**Figure 4A**). Increasing levels of IL-17A, IFN-γ, and IL-15 were all associated with increasing post-vaccination titers against A/Kansas/2017 (**Figures 4B–G**), and they were also individually associated with increasing titers against A/Brisbane/2018 (**Figures 4D, E**), B/Colorado/2017 (**Figures 4F, G**), and B/Phuket/2013 (**Figures 4B, C**), respectively. In contrast to the negative association of Day 0 eotaxin with serological response to vaccination, increasing eotaxin at Day 7 was positively associated with post-vaccination titers against B/Colorado/2017 (**Figures 4H, I**). Additionally, as observed in the Day 7 analysis above, increasing sCD40L was negatively associated with fold-change in titers against A/Brisbane/2018.

## Discussion

This study identified that pre- and post-vaccination plasma analytes correlated with serological response to influenza vaccination. While age was shown to be the strongest correlate of pre-vaccination HAI titers, as has been reported previously, plasma analytes were more strongly associated with changes in titers post-vaccination. Pre-vaccination eotaxin (CCL11) and IL-10 were negatively associated with serological response to vaccination, while MDC (CCL22) was positively associated with serological response. Increasing levels of NK and T cell-associated cytokines IFN-γ, IL-17A, and IL-15 post-vaccination were all positively associated with serological response to vaccination, while the opposite was observed for sCD40L.

Prior to vaccination and also at Day 7 post-vaccination, this study found that eotaxin (CCL11) and MDC (CCL22) were negatively and positively correlated with serological response to vaccination, respectively. The association was the strongest for titers against B/Phuket/2013, which had the highest pre-vaccination titers in this study. CCL11 is a Th2-induced chemokine that promotes the recruitment of eosinophils, basophils, mast cells, and Th2 lymphocytes (16). CCL22 can also promote the recruitment of Th2 lymphocytes (17), and it is secreted during inflammation and can act upon monocytes and NK cells (17, 18). Both chemokines are upregulated during the *in vitro* influenza virus infection of epithelial cells and dendritic cells (19, 20). However, they have not previously been implicated in response to influenza vaccination. A study of inactivated influenza vaccination in mice found that given alone, the vaccine induced Th2 immune responses (21). However, in the context of Th1 cytokines, namely, high IL-12, the vaccine led to Th2 immune responses and greater heterologous serological immunity. In this study, the levels of pre-vaccination CCL22 were positively correlated with multiple inflammatory proteins, including IP-10 (CXCL10) and TNFα, whereas CCL11 levels were positively correlated with the regulatory chemokine MCP-1 (CCL2). This suggests that high levels of CCL22 pre-vaccination, possibly as part of a negative feedback loop, may be associated with increased systemic inflammation, which has been previously associated with serological response to influenza vaccination (11). Additionally, CCL11 increases with age and correlates with aging-associated senescent phenotypes (22). Therefore, its negative relationship with serological responses in this study may be age-related, although our findings were still significant after adjusting for age. In support of this, plasma IL-10 levels, which also increase with age, were negatively associated with serological response to vaccination. To expand on and validate these findings, future studies should either include a larger number of participants with a wide age distribution or focus on specific age groups to facilitate more careful examination of differences by age.

Post-vaccination, this study found that increasing levels of IFN-γ, IL-17A, and IL-15 were all positively correlated with serological response to vaccination. The association was the strongest for titers against A/Kansas/2017, which showed the greatest increase in titer post-vaccination in this study. All three cytokines are known to be involved in the immune response to influenza infection, primarily through promoting effector and memory T cells. Furthermore, multiple studies have reported increased IFN-γ following influenza vaccination (10, 23, 24), while IL-15 as an adjuvant boosts CD4 T-cell immunity (25). Therefore, it is plausible that increases in these cytokines post-vaccination are indicative of the activation of influenza-specific memory T cells. However, it is also possible that they are associated with NK cell activation. IL-15 boosts NK cell responses to influenza viruses (26, 27), while IFN-γ-producing NK cells can be induced following influenza vaccination (23, 28). Increased levels of IFN-γ following influenza vaccination are positively correlated with the levels of granzyme B (29), which could implicate either NK or T cells. Taken together, this suggests that measuring changes in the plasma levels of IFN-γ and/or IL-15 post-vaccination may be a potential surrogate for the activation of cell-mediated immune responses. Given that these findings were based on changes in plasma cytokines at Day 7 post-vaccination, future studies could potentially expand on these by sampling participants within 1–2 days post-vaccination, closer to the peak of vaccine-induced cytokines.

For both pre- and post-vaccination correlates of serological response to vaccination identified in this study, the strength of the association varied by influenza strain and whether it was or was not incorporated in the most recent study season’s vaccine formulation. Pre-vaccination, plasma analytes were most strongly associated with titers against B/Phuket/2013, a strain of the Yamagata lineage. In contrast, post-vaccination, plasma analytes were more strongly associated with titers against A/Kansas/2017, an H3N2 strain. Another study similarly found that the relationship between serological response to influenza vaccination and pre-vaccine cytokines varied by strain (11), with IL-6 in that study being most strongly associated with immune responses against H3N2. While H1N1 remains the most commonly detected strain annually in the United States, H3N2 strains have been associated with a much greater disease burden in the last decade (30). This suggests that immunity against H3N2 strains is more variable across the US population than immunity against H1N1 strains, with host immune factors, such as systemic chemokine and cytokine levels, contributing to this. It may also be related to individual-level variation in exposure to influenza viruses. For example, older individuals may have a longer and more diverse history of exposure to H1N1 strains that were historically, pre-21st century, a greater cause of morbidity. In support of this, titers against the H3N2 strain A/Kansas/2017 had the weakest relationship with age in this study. Additionally, while this study was underpowered to split our analysis by age group, the previously published association of pre-vaccination IL-6 levels with serological responses highlighted earlier was much stronger in younger individuals. Therefore, while strain-specific associations may be due to inherent differences in immunogenicity or virulence, they are as likely to be driven by differences in prior exposure to influenza.

In summary, plasma chemokines and cytokines measured pre- or early post-influenza vaccination have utility as correlates of serological responsiveness. Notably, increasing levels of IFN-γ and IL-15 post-vaccination may indicate the activation of cell-mediated immunity by NK cells and/or T cells. Future studies could build on these observations by focusing on the potential strain specificity, and therefore seasonal variability, of these associations and how age, history of influenza exposure, and post-vaccination timing of sampling may confound findings.

## Data Availability

The datasets presented in this study can be found in online repositories. The names of the repository/repositories and accession number(s) can be found below: https://www.niaidcivics.org/.
