# Supplementary material for: Elevated NK and T cell-associated cytokines in plasma are associated with serological response to influenza vaccination
Source: Front Immunol. 2025 Aug 13;16:1662942. doi: 10.3389/fimmu.2025.1662942 (PMC12380862; doi:10.3389/fimmu.2025.1662942)
Supplement: Supplementary file 1 [file DataSheet1.docx]

**Elevated NK and T cell associated cytokines in plasma associate with serological response to influenza vaccination**

Authors: Harry Pickering^1^, Michael A. Carlock^2^, Monica Cappelletti^1^, David W. Gjertson^1^, Ted M. Ross^2,3,4^, Elaine F. Reed^1#^

Affiliations: ^1^David Geffen School of Medicine, University of California Los Angeles, CA; ^2^Center for Vaccines and Immunology, College of Veterinary Medicine, University of Georgia, Athens, GA; ^3^Florida Research and Innovation Center, Cleveland Clinic, Port Saint Lucie, FL; ^4^Department of Infectious Diseases, College of Veterinary Medicine, University of Georgia, Athens, GA

^#^Corresponding author: Elaine F Reed, [ereed@ucla.mednet.edu](mailto:ereed@ucla.mednet.edu)


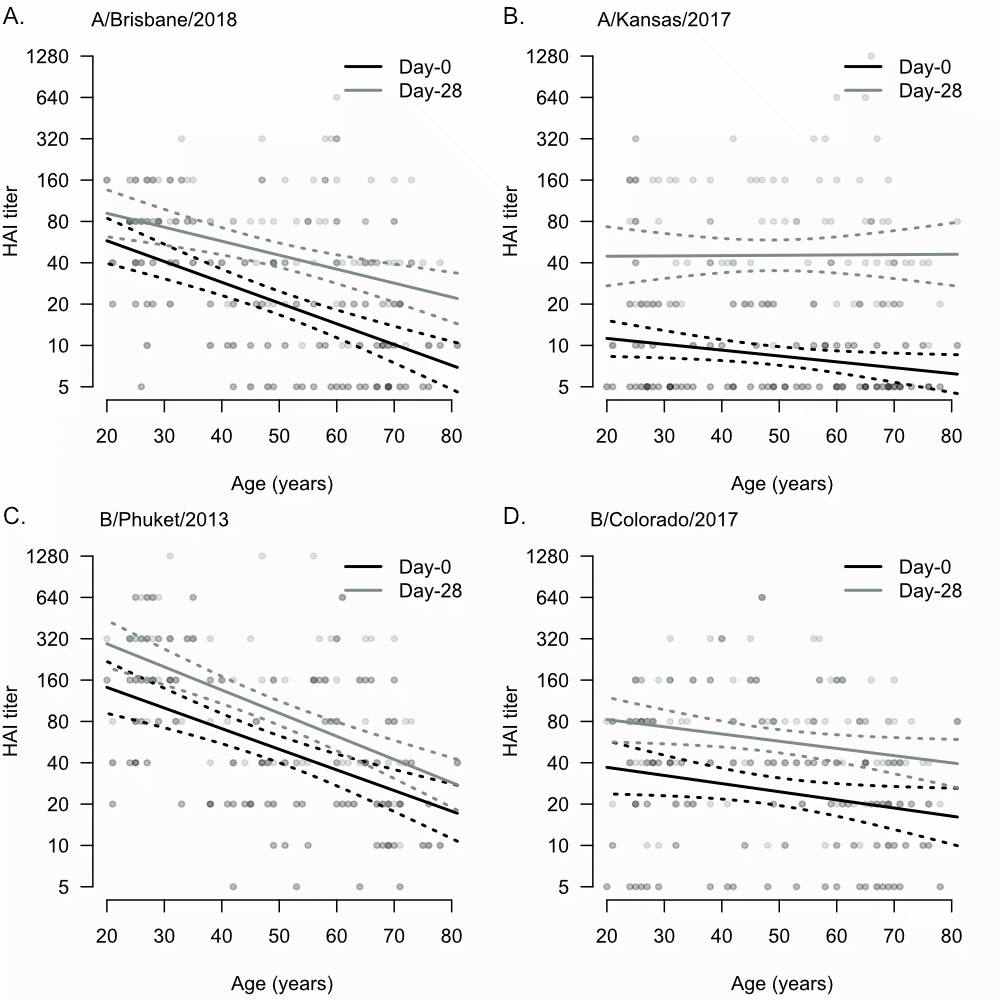


Supplemental Figure 1. Decreasing HAI titers with age.

Visualizing the relationship between participant age and HAI titers against A/Brisbane/2018 (A), A/Kansas/2017 (B), B/Phuket/2013 (C), and B/Colorado/2017 (D). Black and grey line show the linear regression of age on HAI titer at day-0 (pre-vaccination) and day-28 (post-vaccination), respectively. Dotted lines show 95% confidence intervals.


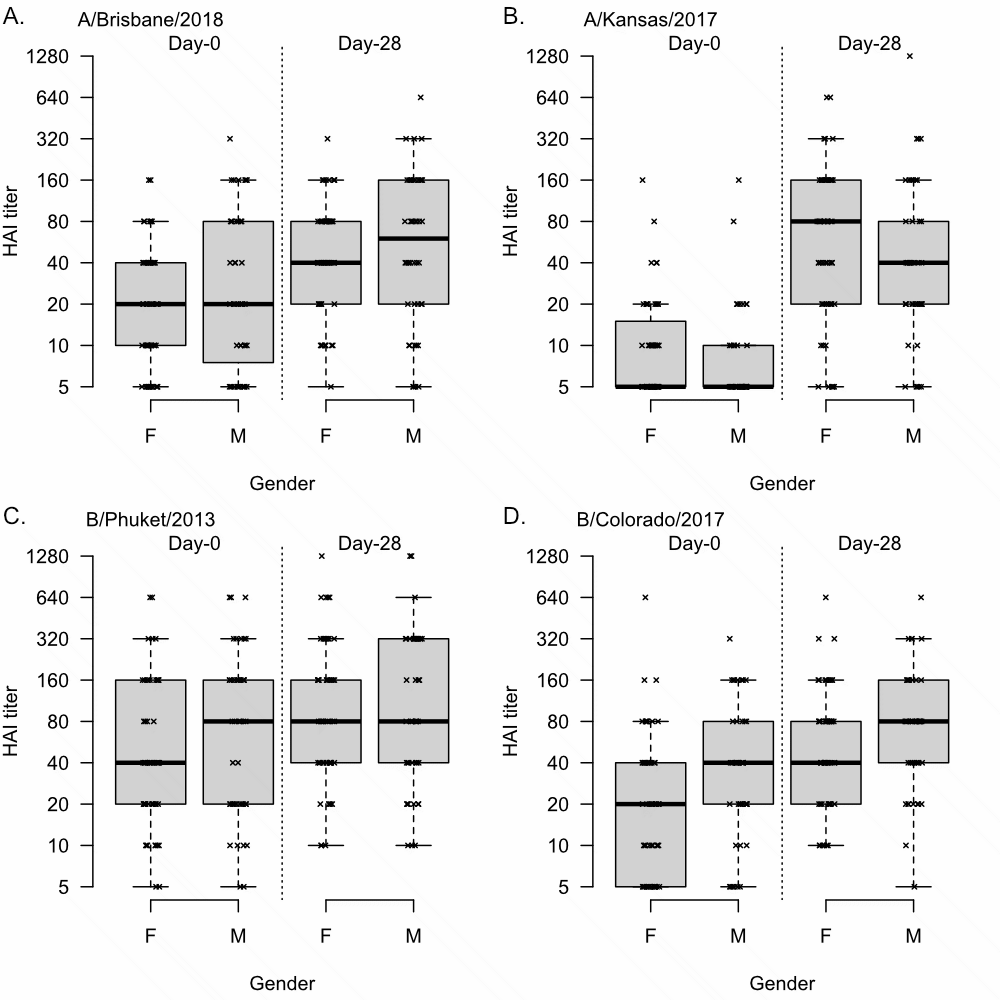


Supplemental Figure 2. Differences in HAI titers by gender.

Visualizing the relationship between participant gender (F=female, M=male) and HAI titers against A/Brisbane/2018 (A), A/Kansas/2017 (B), B/Phuket/2013 (C), and B/Colorado/2017 (D). Boxes show the median and interquartile range (IQR), whiskers were calculated as the 25th percentile minus 1.5 times the IQR and the 75th percentile plus 1.5 times the IQR.


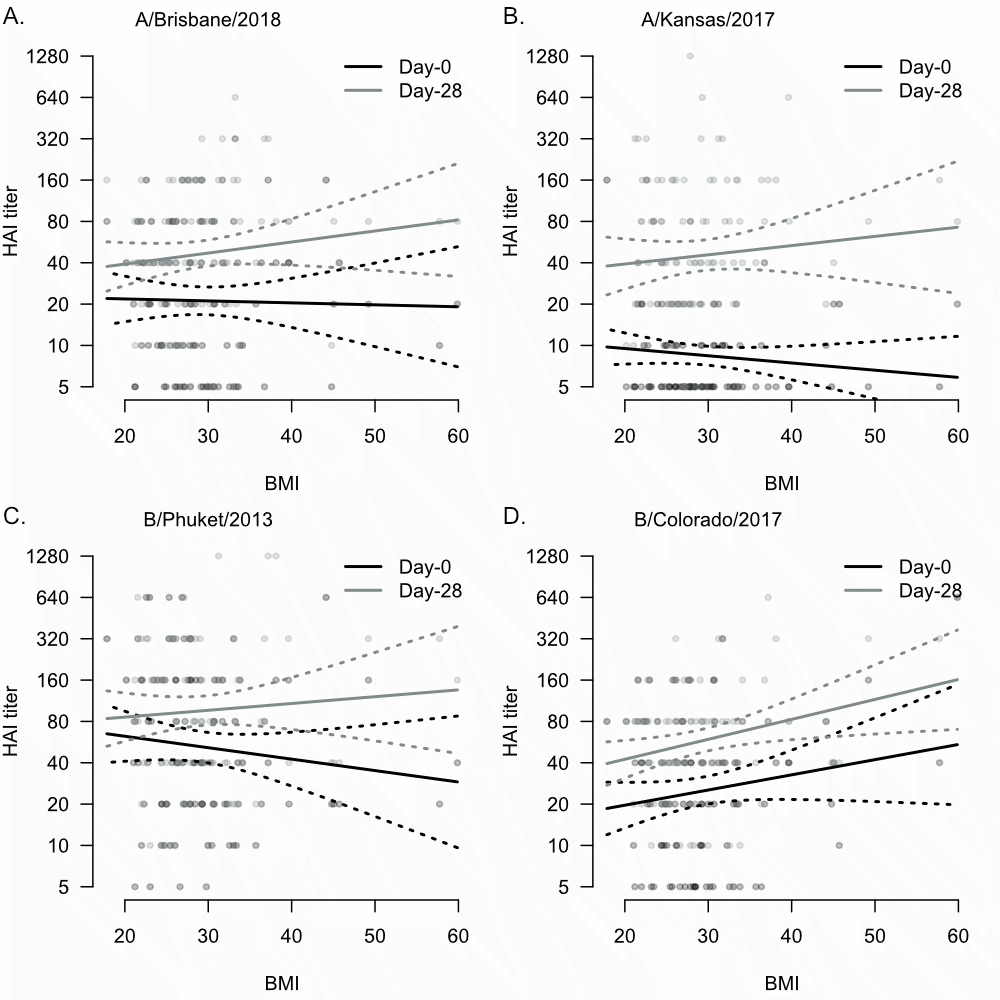


Supplemental Figure 3. Limited association of HAI titers with BMI.

Visualizing the relationship between participant body mass index (BMI) and HAI titers against A/Brisbane/2018 (A), A/Kansas/2017 (B), B/Phuket/2013 (C), and B/Colorado/2017 (D). Black and grey line show the linear regression of age on HAI titer at day-0 (pre-vaccination) and day-28 (post-vaccination), respectively. Dotted lines show 95% confidence intervals.


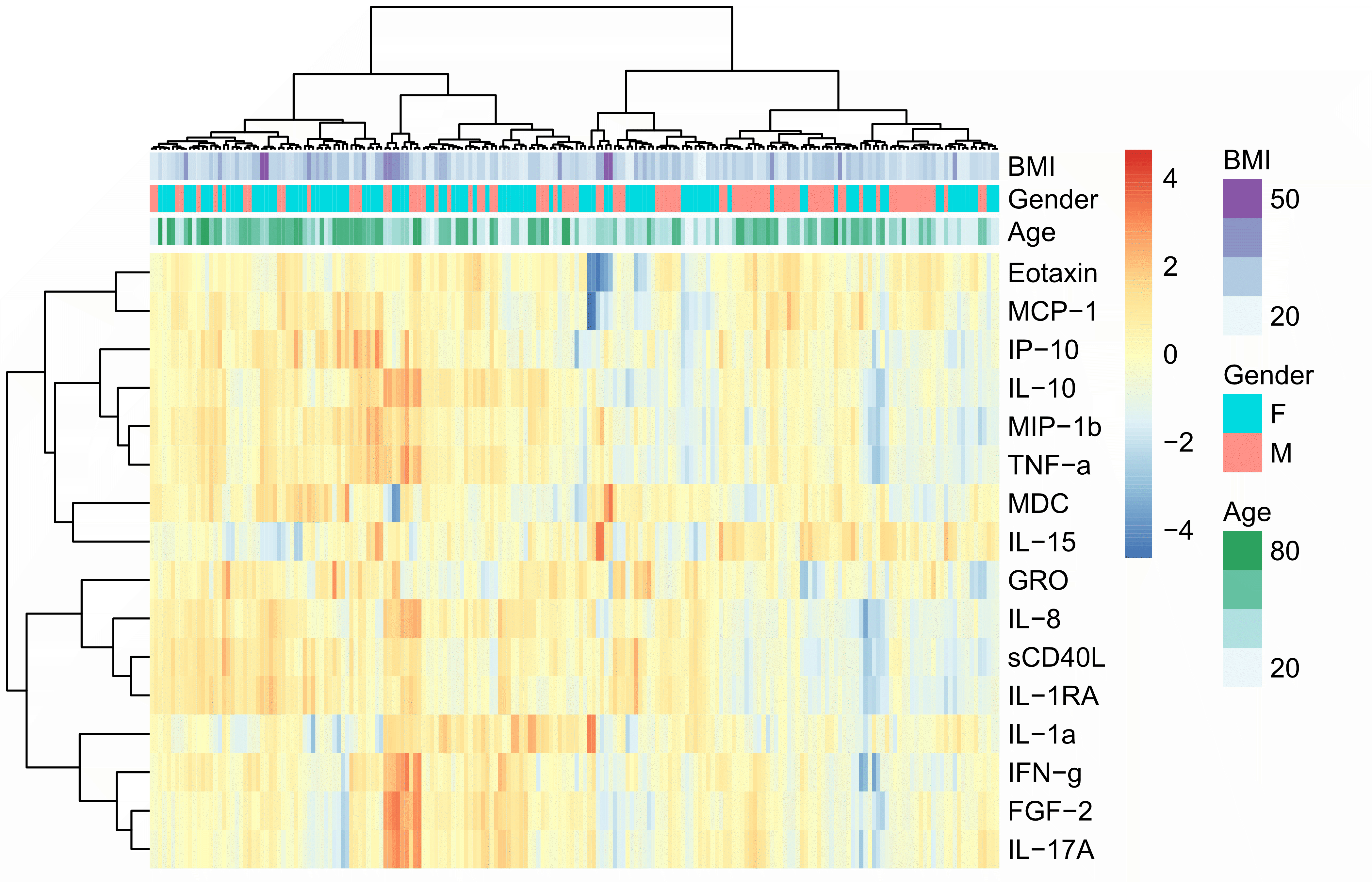


Supplemental Figure 4. Plasma analytes cluster by age and gender

Heatmap of z-score normalized levels of plasma analytes, with key participant demographics overlayed.
